# Value of Twelfth Hour Bilirubin Level in Predicting Significant Hyperbilirubinemia in Preterm Infants

**DOI:** 10.14740/jocmr1639w

**Published:** 2014-03-31

**Authors:** Izi Mayer, Tugba Gursoy, Mutlu Hayran, Secil Ercin, Fahri Ovali

**Affiliations:** a Zeynep Kamil Maternity and Children’s Research and Training Hospital, Istanbul, Turkey; b Hacettepe University Faculty of Medicine Preventive Oncology, Ankara, Turkey

**Keywords:** Hyperbilirubinemia, Neonate, Preterm, Phototherapy

## Abstract

**Background:**

As hyperbilirubinemia is a significant cause of brain injury, it is important to predict the cases who are at risk. Data for preterm infants are scarce. The aim of this study is to predict significant hyperbilirubinemia in preterm infants by measuring capillary bilirubin at 12th hour of life.

**Methods:**

One hundred and fifty neonates born ≤ 35 weeks were included in the study. They were categorized into two groups according to their birth weights (group 1: 1,000 - 1,499 g; group 2: 1,500 - 2,000 g). Their bilirubin levels were measured at 12th hour and daily thereafter for 5 days. Risk nomograms were generated based on their bilirubin measurements and postnatal ages. On the age-specific percentile-based nomogram, the zone above the 90th percentile was determined as high risk and those below the fifth percentile as low risk. Infants who had bilirubin levels over the limits defined according to their postnatal ages and birth weights were accepted to have significant hyperbilirubinemia and received phototherapy and predictive value of the 12th hour bilirubin was asssessed.

**Results:**

Fifty-four of 57 infants (94.7%) in group 1 and 75/93 infants (80.7%) in group 2 received phototherapy. Capillary bilirubin levels of 3.55 mg/dL and 4.55 mg/dL for group 1 and group 2 measured at the 12th hour of life had the highest sensitivity, negative and positive predictive value to predict the neonates who will develop significant hyperbilirubinemia.

**Conclusion:**

Bilirubin levels of preterm infants should be monitored closely. More attention should be paid to the ones who had 12th hour bilirubin level above the cutoff values.

## Introduction

Neonatal jaundice is a common disorder, with more than half of all newborns being affected in the first 3 - 5 postnatal days [1, 2]. Hyperbilirubinemia, when excessive, can lead to potentially irreversible bilirubin-induced neurologic dysfunction. Therefore, early identification of newborn infants at risk for developing severe hyperbilirubinemia has become a public health issue.

In infants at or greater than 35 weeks gestational age (GA), universally accepted guidelines based upon an age-spesific, percentile-based total bilirubin (TB) nomogram are used to decide when to initiate phototherapy (PT) and exchange tranfusion to prevent severe hyperbilirubinemia and bilirubin-induced neurologic dysfunction [3].

Several investigators have tried to find a simple marker to predict severe postnatal hyperbilirubinemia in newborns [2, 4-9]. Some of them used a blood bilirubin determination at an age of 6 up to 24 h to predict the subsequent course of hyperbilirubinemia [5-7], whereas others determined the predictive value of later (> 24 h) bilirubin measurements [2, 8, 9]. However, in infants ≤ 35 GA, similar guidelines are not available, despite observational evidence that suggests preterm infants ≤ 35 GA are more susceptible to bilirubin-induced neurologic dysfunction at lower TB levels than more mature infants.

We aimed to prospectively determine the critical serum TB level to predict significant hyperbilirubinemia in ≤ 35 GA preterm newborns based on serum bilirubin measurements at 12th hour of life.

## Material and Methods

This study was performed in the Neonatal Intensive Care Unit of Zeynep Kamil Maternity and Children’s Training and Research Hospital, Istanbul. The study was approved by the local ethical committe and written informed consent was obtained from the parents. Infants at or less than 35 weeks of GA were included. Infants were categorized into two groups according to their body weights (group 1: 1,000 - 1,499 g and group 2: 1,500 - 2,000 g). Their capillary bilirubin (CB) levels were measured at 12th hour of life and daily thereafter for 5 days. Infants who had bilirubin levels over the limits defined according to their postnatal ages and birth weights were accepted to have significant hyperbilirubinemia and received PT treatment. These infants were dropped from the follow-up. Risk nomograms were generated based on their bilirubin measurements and postnatal ages.

Delivery route, GA, birth weight, Apgar score, mother’s and infant’s blood type, peripheral blood smear, hemoglobin and hematocrit levels were recorded. Infants with sepsis, meningitis, major congenital malformation, perinatal asphyxia, acidosis, blood group incompatibility, positive direct antiglobulin test, glucose-6-phosphate dehydrogenase deficiency, traumatic birth and cephal hematoma, and infants who are small and large for GA infants were excluded from the study. PT was initiated according to the criteria described previously [10].

### Statistical analysis

Data were evaluated by using the SPSS 13.0 for Windows. Nonhomogeneously distributed variables were compared by using Mann-Whitney U test, and Chi-square was used for skewed variables.

The value of 12th hour CB which will predict, with reasonable accuracy, the preterms at risk of subsequent hyperbilirubinemia was determined using receiver operating characteristic (ROC) curve analysis. The sensitivity, specifitiy, positive predictive value (PPV) and negative predictive value (NPV) were calculated.

## Results

One hundred and fifty preterm infants whose GA was ≤ 35 weeks and who had birth weights between 1,000 and 2,000 g were prospectively enrolled in the study. There were 57 infants in group 1 and 93 infants in group 2. The clinical and laboratory features of groups are shown in Table 1.

**Table 1:** The Clinical and Laboratory Features of Groups

	Group 1	Group 2	P
Gestation age (weeks) (mean ± SD)	30.1 ± 2.4	32.8 ± 1.4	< 0.001
Birth weight (g) (mean ± SD)	1,213.8 ± 153.5	1,738.6 ± 153.6	< 0.001
Hematocrit (%) (mean ± SD)	50 ± 5.7	52.9 ± 5.3	0.002
Hemoglobin (g/dL) (mean ± SD)	16.9 ± 1.9	17.9 ± 1.9	0.001
Apgar (fifth minute) (mean ± SD)	7.9 ± 0.9	8.2 ± 0.8	0.027
F/M	33/24	44/49	> 0.05
NSVD/CS	8/49	25/68	> 0.05

CS: Cesarean section; F: female; M: male; NSVD: normal spontaneous vaginal delivery.

In group 1, two infants developed severe hyperbilirubinemia at 12th hour of life and since PT was initiated, they were excluded from the study. All infants in group 1 excluding three infants (5.3%) received PT. Clinical and laboratory features of patients in group 1 according to their PT treatment are shown in Table 2.

**Table 2:** The Clinical and Laboratory Features of Patients in Group 1

	PT (-) (n = 3)	PT (+) (n = 54)
Gestational age (weeks) (mean ± SD)	32.4 ± 2	30 ± 2.3
Birth weight (g) (mean ± SD)	1,221.7 ± 228.3	1,213.4 ± 151.4
Hematocrit (%) (mean ± SD)	49.6 ± 2.6	50.1 ± 5.9
Hemoglobin (g/dL) (mean ± SD)	17.2 ± 0.6	16.9 ± 2
Apgar (fifth minute) (mean ± SD)	8 (8-8)	8 (8-9)
F/M	2/1	31/23
NSVD/CS	0/3	8/46

CS: Cesarean section; F: female; M: male; NSVD: normal spontaneous vaginal delivery; PT: phototherapy.

None of patients in group 2 needed PT at 12th hour of age. Eighteen infants (19.3%) did not receive any PT in group 2. Comparisons of clinical and laboratory features of patients in group 1 according to their PT treatment are shown in Table 3.

**Table 3:** The Clinical and Laboratory Features of Patients in Group 2

	PT (-) (n = 18)	PT (+) (n = 75)	P
Gestational age (weeks) (mean ± SD)	33.1 ± 1.1	32.7 ± 1.4	0.79
Birth weight (g) (mean ± SD)	1,702.8 ± 150.1	1,747.2 ± 154.2	0.41
Hematocrit (%) (mean ± SD)	52.2 ± 4.1	53.1 ± 5.6	0.86
Hemoglobin (g/dL) (mean ± SD)	17.9 ± 1.5	17.9 ± 2	0.74
Apgar (fifth minute) (mean ± SD)	8 (8-9)	8 (8-9)	0.48
F/M	12/6	32/43	0.06
NSVD/CS	2/16	23/52	0.12

CS: Cesarean section; F: female; M: male; NSVD: normal spontaneous vaginal delivery; PT: phototherapy.

No long-term complications of hyperbilirubinemia were seen in either group. TB bilirubin levels of infants are compared in Table 4.

**Table 4:** Daily Capillary Bilirubin Levels of Both Groups

	n	Bilirubin (mg/dL ± SD)	P
12th hour			0.85
Group1	57	4.5 ± 0.8	
Group 2	93	4.7 ± 0.8	
24th hour			0.76
Group1	55	6.3 ± 1.4	
Group 2	93	6.6 ± 1.4	
48th hour			0.016
Group1	35	8.1 ± 1.4	
Group 2	87	8.9 ± 1.7	
72th hour			< 0.001
Group1	9	7.6 ± 1.3	
Group 2	56	9.9 ± 1.8	
96th hour			0.02
Group1	7	8.3 ± 1.9	
Group 2	34	10.2 ± 1.9	
120th hour			0.46
Group1	5	8.2 ± 2.9	
Group 2	27	10.0 ± 2.2	

**Figure 1:**
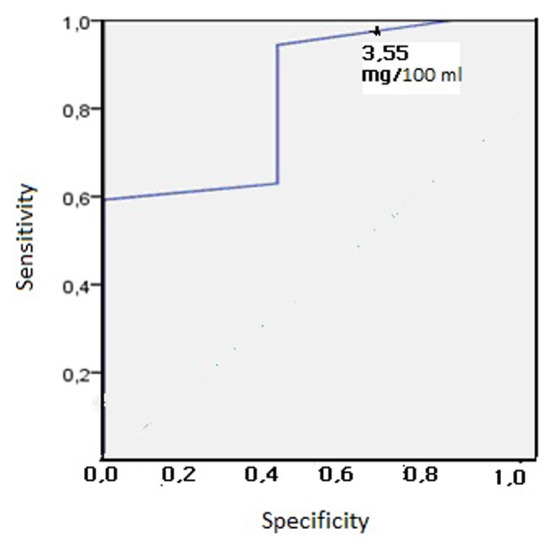
Receiver operating characteristic curve analysis in group 1 (birth weight: 1,000 - 1,499 g) (cutoff: 3.55 mg/dL).

**Figure 2:**
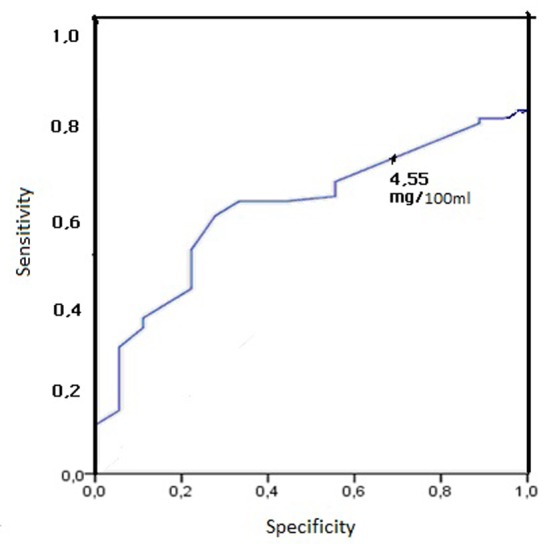
Receiver operating characteristic curve analysis in group 2 (birth weight: 1,500 - 2,000 g) (cutoff: 4.55 mg/dL).

ROC curve analysis was used to determine the cutoff value of 12th hour serum bilirubin level to predict which preterm infants likely develop significant hyperbilirubinemia in both groups (Fig. 1, 2).

First day serum bilirubin of 3.55 mg/dL in group 1 was determined to have the highest sensitivity (94.4%). This critical bilirubin level had a very high PPV (98.1%) and a fairly low NPV (40%) (Fig. 1). Similarly, a first day serum bilirubin of 4.55 mg/dL in group 2 was determined to have the highest sensitivity (70.7%). This critical bilirubin level had a very high PPV (91.4%) and a fairly low NPV (37.1%) (Fig. 2).

**Figure 3:**
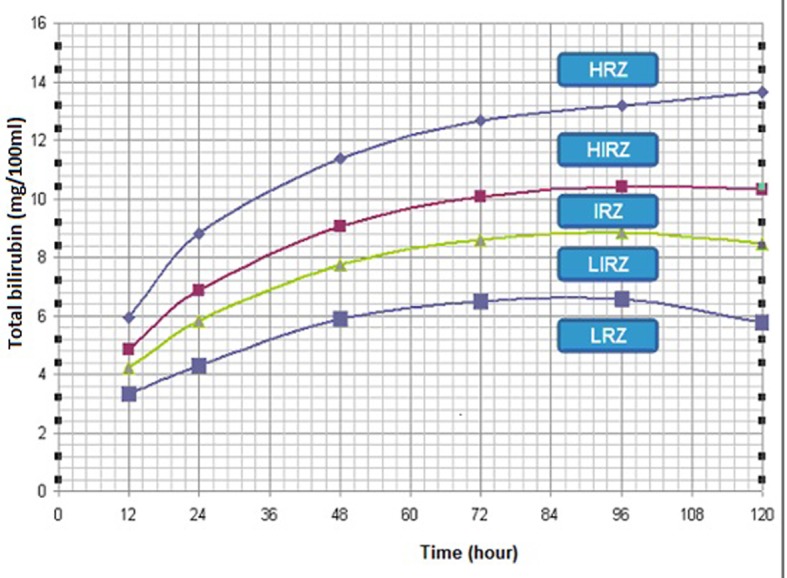
Risk nomogram prepared from total capillary bilirubin levels obtained in the first 5 postnatal days of the infants whose birth weights ranged between 1,000 and 2,000 g (150 patients). HRZ: high risk zone (> 95%); HIRZ: high intermediate risk zone (60-95%); IRZ: intermediate risk zone (30-60%); LIRZ: low intermediate risk zone (5-30%); LRZ: low risk zone (< 5%).

On the hour (age)-specific percentile-based nomogram, the zone above the 90th percentile was determined as high risk and those below the 35th percentile as low risk (Fig. 3).

Every 1 mg/dL increase of CB at 12th hour resulted in an increase in PT requirement by 1.77 times (CI: 1.40 - 2.27). Newborns in group 1 received 2.87 (CI: 2.0 - 4.2) times more PT than the newborns in group 2. Risk of indirect hyperbilirubinemia increased 1.1 (CI: 1.02 - 1.2) times with every 100 g decrement in birth weight and 1.13 (CI: 1.02 - 1.26) times with every one week decrement in GA.

Because of limited number of patients in both groups, risk nomogram of CB for infants in the first 5 postnatal days was not analyzed separately (Fig. 3).

## Discussion

Hyperbilirubinemia in preterm infants is more prevalent, severe and protracted than that in term infants because of increased immaturity of the red blood cells, liver and gastrointestinal tract in preterm infants [11]. While hyperbilirubinemia (total serum bilirubin (TSB): 1.0 mg/dL) occurs in nearly all infants, significant hyperbilirubinemia (TSB: > 12.9 mg/dL) and excessive hyperbilirubinemia (TSB values above the 95th percentile for age in hours) occur in only 5% to 6% of the healthy newborn population [9, 12-14]. Moderate to severe hyperbilirubinemia (> 75th percentile for age in hours) in the newborn usually peaks between 3 and 7 days of age [1, 9].

The major complication of an elevated TB is BIND. Acute bilirubin encephalopathy is the acute and reversible form of BIND, while kernicterus is the chronic and irreversible form of BIND. Preterm infants, compared to term infants, appear to be more vulnerable to BIND at lower TB levels.

Although various parameters, including cord blood bilirubin value [1], mesurements of end tidal carbonmonoxide and serum TB [15] or first day serum bilirubin measurements [6] have been studied to predict the development and severity of a subsequent hyperbilirubinemia, it remains difficult to anticipate because none of these tests have high predictive value. The data for preterm infants are even more inadequate.

Our hypothesis was that in preterms, a high serum bilirubin level at 12th hour of life, would predict a high peak level subsequently. Our study has implied that this hypothesis may be true. We have determined a cutoff value of 3.55 mg/dL in group 1 (1,000 - 1,499 g) and 4.55 mg/dL in group 2 (1,500 - 2,000 g) for the prediction of significant hyperbilirubinemia.

Bhutani et al tested the same hypothesis in a large cohort with term and near-term infants [9]. They proved that infants who develop hyperbilirubinemia have serum bilirubin levels which are in higher percentiles soon after birth. The authors created percentile charts of serum bilirubin levels at different postnatal ages in near-term and term infants whose direct coombs test was negative. They found that 6.1% of the neonates had predischarge serum bilirubin in the ≥ 95th percentile range and 32.1% of these neonates subsequently developed significant hyperbilirubinemia. In a similar study by Alpay et al [6], a first day serum bilirubin of 6 mg/dL was determined to have the highest sensitivity (90%). This critical bilirubin level had a very high NPV (97.9%) and a fairly low PPV (26.2%).

In another study by Awasthi et al [16], a value of 3.99 mg/dL was found to have a sensitivity of 64.2% and 67.4% for subsequent requirement of PT. However, there were major flaws in the study. The cutoff value was not determined using ROC analysis but rather the mean serum bilirubin at 18 - 24 h was used as the cutoff for developing the “prediction test”. Moreover, complete follow-up was conducted in infants who stayed in the hospital either for neonatal illness or some maternal reason. More than 50% of the neonates who were healthy, thus discharged early, were not studied. In a study by Agarwal et al [7] the predictive ability of TSB value of 6 mg/dL at 24 ± 6 h of life was evaluated and a sensitivity of 95%, specificity of 27.2% and NPV of 99.3% were determined. In the present study, the study population was composed of preterm babies. PPVs were high for both groups; however, NPVs were lower than the previous studies. This difference may be due to the premature population of our study as they need much more PT than the term infants.

On the hour (age)-specific percentile-based nomogram, the zone above the 90th percentile was determined as high risk and those below the 35th percentile as low risk (Fig. 3). These values are somewhat similar to those found in term infants in other studies, but a universal approach for all neonates could not be made and preterm infants should be evaluated separately.

Though the serum bilirubin measurements at 12th hour of life is useful in predicting the postnatal bilirubin values in preterm newborns, most of the infants had to receive PT, so this might have obscured the value of the predictivity of the 12th hour bilirubin level and this was the limitation of our study.

Laboratory examination of preterm infants rather than clinical observation should be preferred as we cannot rely on skin colors to predict bilirubin levels. Twelfth hour capillary total biluribin levels, over the cutoff values, have a good prediction of hyperbilirubinemia and helpful in reducing the risk of BIND or kernicterus by starting early treatment. Unfortunately low NPV of our cutoff values should alert the clinicians to monitor preterm babies clinically more closely.

Our results should be verified by larger trials in preterm infants before a solid recommendation can be made.

## References

[1] Knupfer M, Pulzer F, Gebauer C, Robel-Tillig E, Vogtmann C (2005). Predictive value of umbilical cord blood bilirubin for postnatal hyperbilirubinaemia. Acta Paediatr.

[2] Seidman DS, Ergaz Z, Paz I, Laor A, Revel-Vilk S, Stevenson DK, Gale R (1999). Predicting the risk of jaundice in full-term healthy newborns: a prospective population-based study. J Perinatol.

[3] (2004). American Academy of Pediatrics Subcommittee on H. Management of hyperbilirubinemia in the newborn infant 35 or more weeks of gestation. Pediatrics.

[4] Stevenson DK, Fanaroff AA, Maisels MJ, Young BW, Wong RJ, Vreman HJ, MacMahon JR (2001). Prediction of hyperbilirubinemia in near-term and term infants. Pediatrics.

[5] Newman TB, Liljestrand P, Escobar GJ (2002). Jaundice noted in the first 24 hours after birth in a managed care organization. Arch Pediatr Adolesc Med.

[6] Alpay F, Sarici SU, Tosuncuk HD, Serdar MA, Inanc N, Gokcay E (2000). The value of first-day bilirubin measurement in predicting the development of significant hyperbilirubinemia in healthy term newborns. Pediatrics.

[7] Agarwal R, Kaushal M, Aggarwal R, Paul VK, Deorari AK (2002). Early neonatal hyperbilirubinemia using first day serum bilirubin level. Indian Pediatr.

[8] Bhutani VK, Johnson LH (2000). Managing the assessment of neonatal jaundice: importance of timing. Indian J Pediatr.

[9] Bhutani VK, Johnson L, Sivieri EM (1999). Predictive ability of a predischarge hour-specific serum bilirubin for subsequent significant hyperbilirubinemia in healthy term and near-term newborns. Pediatrics.

[10] Halamek LP, Stevenson DK, Fanaroff AA, Martin RJ (2002). Neonatal jaundice and liver disease. Neonatal-perinatal medicine diseases of the fetus and infant. Volume 2.

[11] Watchko JF, Maisels MJ (2003). Jaundice in low birthweight infants: pathobiology and outcome. Arch Dis Child Fetal Neonatal Ed.

[12] Cashore WJ (1991). Neonatal hyperbilirubinemia. N Y State J Med.

[13] Gartner LM (1992). Management of jaundice in the well baby. Pediatrics.

[14] Gourley GR (1997). Bilirubin metabolism and kernicterus. Adv Pediatr.

[15] Stevenson DK, Fanaroff AA, Maisels MJ, Young BW, Wong RJ, Vreman HJ, MacMahon JR (2001). Prediction of hyperbilirubinemia in near-term and term infants. J Perinatol.

[16] Awasthi S, Rehman H (1998). Early prediction of neonatal hyperbilirubinemia. Indian J Pediatr.

